# Effect of Empagliflozin on Serum Ferritin and Its Relationship With Inflammatory Markers in Prediabetic and Diabetic Patients

**DOI:** 10.1155/jdr/8835829

**Published:** 2025-03-20

**Authors:** Mojgan Sanjari, Narges Sadeghi, Azade Ramezannezhad, Zohre Safi, Ahmad Naghibzadeh-tahami, Ladan Amirkhosravi

**Affiliations:** ^1^Endocrinology and Metabolism Research Center, Kerman University of Medical Sciences, Kerman, Iran; ^2^Physiology Research Center, Institute of Neuropharmacology, Kerman University of Medical Sciences, Kerman, Iran; ^3^Modeling in Health Research Center, Institute for Futures Studies in Health, Kerman University of Medical Sciences, Kerman, Iran; ^4^Department of Biostatistics and Epidemiology, Kerman University of Medical Sciences, Kerman, Iran

**Keywords:** CRP, ferritin, interleukin-6, iron metabolism, sodium–glucose cotransporter-2 inhibitors, uric acid

## Abstract

**Background:** This research is aimed at evaluating the effects of the SGLT2 inhibitor empagliflozin on inflammatory markers, some iron metabolism markers, and their interrelationships before and after using empagliflozin.

**Methods:** A quasiexperimental study was conducted on 44 prediabetic and Type 2 diabetic patients aged 18–65 years. The participants were among those treated at the clinic affiliated with Kerman Medical Sciences University between 2022 and 2023. The study included diabetic patients with HbA1c levels of 0.5%–1% higher than the therapeutic target who were not using blood sugar control medication. Each patient received a daily dose of 10 mg of empagliflozin for 3 months. Changes in serum levels of iron, total iron-binding capacity (TIBC), ferritin, transferrin saturation, inflammatory markers IL-6, C-reactive protein (CRP), and uric acid were measured before and 3 months after commencing empagliflozin.

**Results:** Three months after starting empagliflozin, the mean FPG and Hb A1c levels showed a drop (*p* < 0.05). The serum ferritin level decreased, and TIBC increased significantly (*p* < 0.05) following empagliflozin treatment. Additionally, the serum levels of CRP (*p* < 0.05), IL-6 (*p* < 0.001), and uric acid (*p* < 0.001) declined. Analysis of the correlation between serum ferritin level and IL-6 and uric acid before and after empagliflozin use revealed a positive correlation between serum ferritin and IL-6 (*p* = 0.04) and uric acid (*p* = 0.03). However, no significant correlation was observed between the change in ferritin and CRP levels (*p* = 0.22).

**Conclusion:** Following empagliflozin treatment, serum levels of ferritin and inflammatory markers interleukin-6, CRP, and uric acid declined, indicating a significant relationship between SGLT2 inhibition, inflammation, and iron metabolism. Furthermore, the correlation between ferritin and inflammatory markers suggests that reduced ferritin levels may result from reduced inflammation.

**Trial Registration:** ClinicalTrials.gov identifier: IRCT20090317001774N10

## 1. Introduction

Ferritin is a protein that stores iron in the body. When iron levels are low, ferritin levels decrease [1]. Diabetes and iron metabolism are closely connected, with a two-way relationship between glucose regulation and iron levels. Indicators of iron metabolism (e.g., transferrin and ferritin) can directly affect the occurrence and progression of Type 2 diabetes (T2D), with higher serum ferritin levels being a risk factor for T2D [2]. Elevated serum ferritin levels are associated with an increased risk of prediabetes, possibly due to ferritin's contribution to low-grade chronic inflammation and the oxidation of LDL [3]. However, further investigation is needed to understand the exact mechanisms and the potential role of iron deficiency in prediabetes. Ferritin has been found to play a crucial role in regulating inflammation. Furthermore, elevated ferritin levels may serve as an indicator of inflammation [4, 5], which plays a role in the onset of prediabetes and T2D.

Diabetes and inflammation are closely linked, with inflammation playing a significant role in the development and progression of diabetes [6]. In T2D, chronic inflammation increases insulin resistance and disrupts glucose metabolism. The release of inflammatory cytokines like TNF-alpha and IL-6 can impair insulin signaling and contribute to the development of insulin resistance [7]. Furthermore, chronic inflammation in T2D is associated with the development of microvascular complications, such as diabetic nephropathy, neuropathy, and retinopathy. These complications are characterized by the production of proinflammatory cytokines and the activation of immune cells, leading to tissue damage and dysfunction [6].

Sodium–glucose transporter inhibitors (SGLT2) such as empagliflozin, canagliflozin, and dapagliflozin represent the newest class of hypoglycemic medications. SGLT2 inhibitors have become well-known for their beneficial effects on glycemic control, reducing cardiovascular risk, slowing the progression of chronic kidney disease (CKD), and lowering the risk of kidney failure in individuals with T2D [8]. Additionally, these medications help normalize lipid metabolism, preventing diabetes-related dyslipidemia and associated diseases caused by abnormal lipid levels [9]. Recent studies suggest that empagliflozin may have additional benefits beyond controlling blood sugar levels, including potential effects on iron metabolism and inflammation [10].

Empagliflozin treatment led to an early increase in plasma erythropoietin levels, higher hematocrit, and reduced ferritin and RBC hemoglobin concentration after 6 months in individuals with T2D mellitus and coronary artery disease [11]. Thiele et al. reported that empagliflozin use in patients with T2D and prevalent atherosclerotic CVD or high CV risk increased red blood cell count and transferrin concentrations. The results showed an ascending trend toward increased erythropoietin levels. In contrast, ferritin, total iron, and transferrin saturation levels decreased [12]. Therefore, the increase in urinary glucose excretion correlated with the induction of erythropoietin in empagliflozin-treated patients. Empagliflozin increased hemoglobin concentrations and hematocrit. This increase is most likely attributable to increased erythropoiesis with augmented iron utilization rather than haemoconcentration [12]. Moreover, empagliflozin slows the progression from prediabetes to diabetes and improves liver lipid metabolism [13]. Additionally, empagliflozin increases insulin sensitivity in the hypothalamus of individuals with prediabetes [14].

SGLT2 inhibitors have been discovered to possess anti-inflammatory properties in different studies [15, 16]. These properties are credited to their capacity to decrease systemic inflammation and oxidative stress, which are linked to cardiovascular issues in individuals with T2D and coronary artery disease [17]. S.A. Tan and L. Tan demonstrated that empagliflozin had superior effectiveness in normalizing elevated IFN-*λ*, TNF-*α*, and IL-6 levels in diabetic patients than canagliflozin [18]. In another study, empagliflozin reduced serum ferritin and uric acid levels more than semaglutide (glucagon-like peptide-1 receptor agonists). Both drugs create only minor changes in some inflammatory markers, such as IL-6 and C-reactive protein (CRP) [19].

The increase in ferritin levels in prediabetes and diabetes patients can be a marker for inflammation [20, 21]. Besides, research has shown increased ferritin concentration in oxidative stress and chronic inflammation [20, 22]. Therefore, investigating factors that contribute to reducing oxidative stress and the inflammatory process is of great importance in patients with diabetes and prediabetes. In this study, we investigated the serum levels of inflammatory markers, some iron metabolism markers, and their relationships before and after using empagliflozin.

## 2. Material and Methods

### 2.1. Subjects

This quasiexperimental study with a before-and-after design was conducted on 44 prediabetic patients or patients with a definite diagnosis of DM. The participants were 18–65 years of age and were among those who were referred to one of the clinics affiliated with Kerman University of Medical Sciences in 2021–2022. They were also a member of the Kerman coronary artery disease risk factors study (KERCADRS). After obtaining the required approval from the Ethics Committee of Kerman University of Medical Sciences (IR.KMU.AH.REC.1402.121), the patients were selected considering the exclusion and inclusion criteria. The study procedures were described to the patients, and a written informed consent form was taken from them to enter the study.

The sample size was estimated according to the research conducted by Thiele et al. [12]. The average ferritin level at the beginning of the study was 181 ± 189. After 3 months of intervention with empagliflozin, this level decreased to 122 ± 148. Using the sample size formula for a paired *T*-Test and the pwr package in R software, the required sample size was calculated with a power of 0.80 and a Type I error rate of 0.05. This calculation indicated that a sample size of 41 would be appropriate, and accounting for a potential drop-out rate of 10%, the final sample size was set at 44. The inclusion criteria were being 18–65 years old, suffering from prediabetes or diabetes with a maximum HbA1c level of 0.5%–1% higher than the appropriate treatment goal of the patient and not taking blood sugar control drugs. In this research, prediabetes and diabetes were defined based on the criteria provided by the American Diabetes Association. Meeting one of the following criteria was considered as having prediabetes: laboratory glucose level of 100–125 mg/dL after 8 h of fasting and a 2-h laboratory glucose level of 140–199 mg/dL (2 h after consuming 75 g of oral glucose). Meeting one or two of the following criteria was considered as having diabetes: glucose level ≥ 126 mg/dL and level of glucose ≥ 200 mg/dL.

The exclusion criteria were a history of suffering from Type 1 diabetes mellitus (T1DM), taking oral or injectable drugs controlling glucose levels (i.e., insulin, biguanides, sulfonylureas, thiazolidinediones, GLP-1 receptor agonists, SGLT2 inhibitors, bromocriptine, or cholestyramine), a history of diabetic ketoacidosis, glomerular filtration rate (GFR) < 30, suffering from underlying diseases that predispose one to acidosis, having liver failure, and taking biotin.

**Figure 1:**
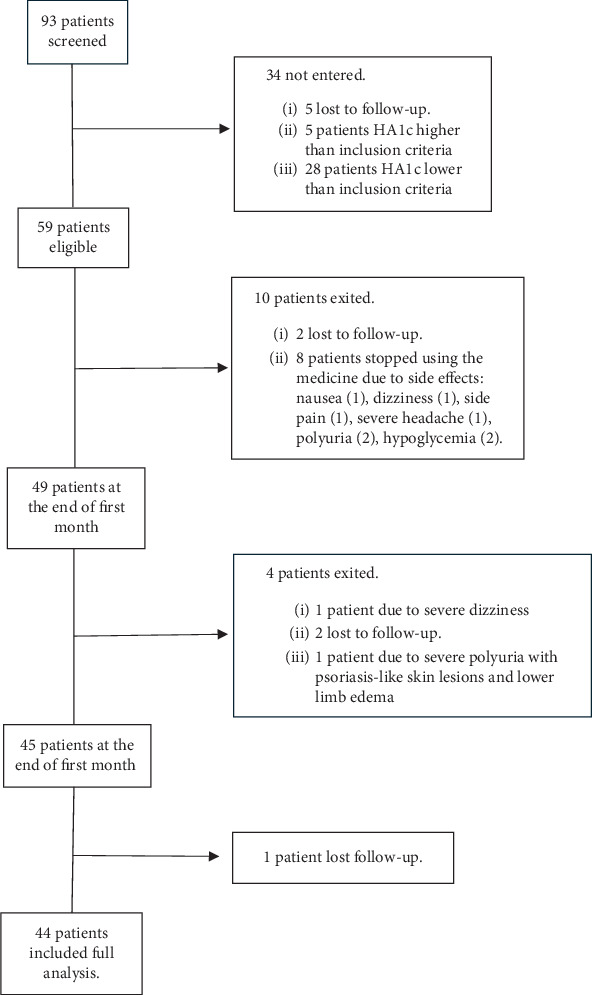
The flowchart for recruiting the study subjects.

In this study, 93 patients were included in the order of their admission. Among all, 34 patients did not meet the inclusion criteria after performing the initial tests and were excluded, 5 of whom did not refer after the initial screening despite making multiple phone calls. Also, 5 and 28 individuals were excluded due to having HbA1c levels higher and lower than the inclusion criterion. Of these participants, 10 patients were excluded during the first month: Two patients were excluded due to nonreferral despite making multiple calls, and eight patients were excluded because of stopping drug consumption due to its side effects (nausea (1 patient), flank pain (1 patient), polyuria (2 patients), hypoglycemia based on the patient's statements and glucometer (2 patients), dizziness (1 patient), and severe headache (1 patient). Out of the remaining 49 patients, 4 patients were excluded from the study during the second month: one person due to severe dizziness, two patients due to lack of cooperation and nonreferral, and one patient due to severe polyuria with psoriasis-like skin lesions and lower limb edema. Also, one patient did not refer after 3 months of receiving the medicine despite making several phone calls. Finally, 44 patients (22 female and 22 male) completed the follow-up period (Figure 1).

### 2.2. Interventions

The patients were asked to go to the clinic laboratory between 8 and 10 a.m. on the specified day after fasting for 12 h to take blood samples. Thus, 10 mL of blood was collected from the patients and stored at −80°C at the Kerman Endocrine and Metabolism Research Center laboratory. Then, all the patients received 10 mg of empagliflozin (manufactured by Dr. Abidi's pharmaceutical company, Iran, under the brand name Gloripa) once daily for 3 months. The drug was prescribed monthly. At the end of each month, the patients were examined in terms of side effects, drug use continuation, height, weight, and blood pressure. Also, at the end of 3 months, the patients were recalled going to the clinic laboratory between 8 and 10 a.m. on the specified day after fasting for 12 h to take blood samples. The blood samples were stored at −80°C. At the end of 3 months, the blood samples were assayed in terms of serum levels of iron, total iron-binding capacity (TIBC), ferritin, transferrin saturation, inflammatory markers IL-6, CRP, uric acid, Cr, fasting plasma glucose (FPG), and HbA1c in the same conditions as before receiving the drug.

### 2.3. Primary Outcomes

The primary outcomes included changes in serum levels of iron, TIBC, ferritin, transferrin saturation, inflammatory markers IL-6, CRP, and uric acid.

### 2.4. Laboratory Analyses

Serum iron level and TIBC were checked by the photometric method, and ferritin was tested by quantitative luminescence method using the kit (Roche company, Germany) with the Cobas device (Germany). Serum IL-6 levels were measured using an enzyme-linked immunosorbent assay kit (Demeditec, Germany). The samples were examined on an automated enzyme-linked immunosorbent assay plate reader (Model No. ELX-80MS, Biotech). CRP was measured by quantitative luminescence method using the kit)Biorex company, Iran) using the Cobas device. Moreover, serum uric acid level was evaluated photometrically by a Selectra E autoanalyzer.

### 2.5. Statistical Analysis

The data were expressed as mean ± standard deviation (SD). Changes in the mean parameters of the study were investigated by the paired samples *t*-test or its nonparametric version, that is, Wilcoxon, considering the normal or nonnormal distribution of the data. Also, a general estimating equation (GEE) was used to investigate the relationship between serum ferritin level and inflammatory markers before and after taking empagliflozin. The significance level was set to 95% and a *p* value of less than 0.05. Analysis of statistics was accomplished using the SPSS software Version 22.

## 3. Result

In this study, the mean age of 44 patients who entered the study was 54.77 years.  Table 1 shows the basic characteristics of patients. According to Table 1, patients' mean weight at the beginning of the study was 77.65 ± 15.97 kg. After receiving empagliflozin, the patients' mean weight significantly decreased by 3 kg (4.056% decrease) (*p* < 0.001). Regarding BMI and waist circumstances, there were no significant differences between before and after empagliflozin use.

The level of FPG and HbA1c after 3 months of empagliflozin use was significantly decreased (*p* < 0.05) (Table 2). The level of serum Cr increased significantly after 3 months of drug use (*p* < 0.001). Also, the GFR level decreased significantly after 3 months of empagliflozin use (*p* < 0.001) (Table 2).

Table 3 shows changes in inflammatory markers and hematopoiesis. The mean serum level of iron at the beginning of the study was 116.27 ± 48.01*  μ*mol/L. However, after receiving empagliflozin, the iron level decreased by 8.83 ± 7.75*  μ*mol/L, which was not statistically significant (*p* = 0.27). The mean level of serum TIBC in the study patients before receiving empagliflozin was 343.79 ± 35.28. Meanwhile, after receiving the drug, its value increased significantly to 355.09 ± 40.56 (*p* < 0.05). The transferrin saturation level decreased after receiving empagliflozin, but this decrease was not statistically significant (*p* = 0.12). The initial study serum ferritin level was 115.47 ± 142.38*  μ*g/L. After taking empagliflozin, it decreased significantly to 94.32 ± 117.68*  μ*g/L (*p* < 0.05).

The initial serum level of CRP was 2.81 ± 3.82. Three months after taking empagliflozin, the CRP level decreased to 2 ± 1.53, which was statistically significant (*p* = 0.02). IL-6 serum level was initially 6.63 ± 17.57. After taking the drug, the level of IL-6 decreased significantly to 6.52 ± 2.21 (*p* < 0.001). The serum uric acid level was 7.1 ± 2.32 at the beginning of the study and before receiving the medication. Three months after taking empagliflozin, the serum uric acid level dropped to approximately 5.66 ± 2.22, which was statistically significant (*p* < 0.001) (Table 3).

### 3.1. Correlation Between Changes in Serum Ferritin Level With Serum Iron Level, TIBC, and Transferrin Saturation Before and After Taking Empagliflozin

Table 4 presents the correlation coefficient between changes in serum ferritin level and other iron parameters from baseline to 3 months by treatment. As can be seen, serum ferritin was positively correlated with iron concentration (*β* = 0.44, *p* < 0.05) and transferrin saturation (*β* = 1.86, *p* < 0.01) (Table 4). A negative correlation between changes in serum ferritin and TIBC was observed before and after empagliflozin use (*β* = −0.82, *p* < 0.001) (Table 4).

### 3.2. Correlation Between Changes in Serum Ferritin and Inflammatory Markers Before and After Using Empagliflozin

Correlation coefficients between changes in serum ferritin level and inflammatory markers from baseline to 3 months by treatment are shown in Table 5. Serum ferritin was positively correlated with IL-6 (*β* = 0.66, *p* = 0.04) and uric acid (*β* = 6.32, *p* = 0.03). Analysis of the correlation between serum ferritin level and CRP before and after using empagliflozin showed that the change in CRP level and ferritin had no significant relationship (*β* = 1.36, *p* = 0.22) (Table 5).

## 4. Discussion

This research examines the effect of empagliflozin consumption on iron metabolism and inflammatory markers in patients with prediabetes and diabetes. The study is aimed at establishing whether the decrease in ferritin is a beneficial outcome of the drug in mitigating inflammation or is due to reduced iron stores in the body. The findings revealed that after 3 months of empagliflozin treatment, there was a significant decrease in serum ferritin levels and a notable increase in Total TIBC. After 3 months of using empagliflozin, there was a significant reduction in serum levels of IL-6, uric acid, and CRP. Additionally, the study revealed a positive significant correlation between changes in ferritin levels and serum iron, TIBC, and transferrin saturation levels. Furthermore, the decrease in serum ferritin levels was also significantly associated with the levels of the inflammatory factors IL-6 and uric acid.

Several studies have shown a strong link between high serum ferritin levels and an increased risk of developing diabetes [23, 24]. Furthermore, a positive correlation was reported between T2D and serum ferritin concentration [25]. An increase in serum ferritin levels is not only considered a risk factor for T2D but is also observed in patients with prediabetes [26]. A systematic study and meta-analysis in 2020 showed that elevated serum ferritin is one of the risk factors for T2D. Also, serum ferritin levels were higher in people with impaired fasting glucose (IFG) than normal [27]. Elevated ferritin is linked to oxidative stress and chronic inflammation, leading to islet beta-cell dysfunction, liver dysfunction, and insulin resistance [20].

In this study, empagliflozin decreased serum ferritin levels and increased TIBC levels. Serum iron and transferrin levels also declined with empagliflozin consumption, which was not statistically significant. Furthermore, a positive correlation was noticed between the reduction of serum ferritin levels and iron and transferrin levels before and after 3 months of empagliflozin use. A negative correlation was observed between serum ferritin level and serum TIBC. In line with the current study, Repo et al. noted that after 3 months of use, empagliflozin reduced serum ferritin levels compared to semaglutide [19]. Koshino et al. reported that dapagliflozin also reduced ferritin and inflammatory markers [28]. Thiele et al., in a study on patients with diabetes, demonstrated that inhibiting SGLT2 increases erythropoietin levels while lowering the levels of saturated transferrin and ferritin [12]. Another study found that dapagliflozin reduced the levels of ferritin, hepcidin, and transferrin while increasing the erythropoietin level in diabetic and obese patients. Besides, it was observed that SGLT2 inhibitors in patients with heart failure decreased hepcidin and ferritin levels and increased transferrin receptor protein [29]. The findings of this study, along with other studies, indicate that inhibition of SGLT2 leads to increased movement of iron from macrophage stores (due to reduced hepcidin) and intracellular sequestration (due to reduced ferritin) [30]. It is recommended to check anemia and iron stores in Type 2 diabetic patients taking empagliflozin.

This research also investigated the change in the serum level of inflammatory markers using empagliflozin in diabetic and prediabetic patients. Inflammation is among the known causes of T2D complications [31]. Insulin resistance and hyperglycemia cause chronic inflammation [32, 33]. Inflammation is highly associated with the progression of diabetic kidney disease and cardiovascular events. Several in vivo and in vitro studies have shown anti-inflammatory effects as the beneficial mechanism of SGLT2 inhibitors [34, 35]. Empagliflozin in diabetes with renal insufficiency improved antioxidative capacity and improved renal function [36]. In the present study, empagliflozin caused a significant decrease in serum levels of IL-6, CRP, and uric acid. In preclinical models of diabetes, SGL2 inhibition reduces hyperglycemia-induced oxidative stress and attenuates interstitial tubular cell inflammation and fibrosis [2]. In agreement with the present study, a crossover clinical trial study concerning canagliflozin and empagliflozin showed no significant decrease in IFN-*γ*, TNF-*α*, and IL-6 levels in the canagliflozin group. When the drug canagliflozin was switched to empagliflozin, the IFN-*γ*, TNF-*α*, and IL-6 levels decreased significantly [18]. A recent study reported that dapagliflozin lowered the inflammatory markers of monocyte chemoattractant protein-1 (MCP-1) and IL-6 [18]. However, Reppo et al. did not observe a meaningful change in classical inflammatory markers CRP and IL-6 levels during 3 months of semaglutide and empagliflozin. One possible reason for the difference between the present study and Reppo's study might be the smaller sample size in the former [19].

Recent scientific evidence describes a bidirectional relationship between glucose and iron metabolism. These relationships are affected by oxidative stress and inflammation. Accordingly, they can strengthen the pathogenic processes that lead to diabetes complications [2]. Diabetes is an inflammatory disease associated with disturbances in iron metabolism [20]. Increased systemic inflammation worsens iron availability partly through increased hepcidin [37]. In the present study, the relationship between serum ferritin levels and inflammatory markers was investigated. The results of the correlation analysis revealed a positive relationship between ferritin level and inflammatory markers IL-6 and uric acid before empagliflozin and 3 months later. In agreement with the present study, a recent study has reported that dapagliflozin reduced ferritin and inflammatory markers, suggesting potentially significant effects of this drug on iron metabolism and inflammation [28]. In Reppo's study, empagliflozin significantly reduced uric acid and ferritin levels (reducing inflammation) [19]. La Grotta et al. also found that patients treated with SGLT-2 inhibitors had lower serum levels of IL-6, fasting insulin, and uric acid than those treated with other diabetes control drugs. In another study, the potential role of uric acid and insulin in mediating the effects of SGLT-2i on IL-6 was investigated. To this end, relevant doses of these two substances (0.5 mM uric acid and 1 nM insulin) in two in vitro models of low-grade inflammation were assessed: monocytes (THP-1) treated with LPS, and human umbilical vein endothelial cells (HUVECs) exposed to elevated levels of glucose. The results revealed that uric acid, in combination with insulin, played a proinflammatory role in monocytes [38]. Meanwhile, uric acid alone had a proinflammatory effect on endothelial cells stimulated by high glucose levels [39].

One of the limitations of this study, the blood samples required for CBC were not collected from patients according to the flow of the original plan. The strengths of this study included the prospective design and the exclusion of patients who were taking other blood sugar control drugs. Also, this research was conducted as a before-and-after study, with subjects serving as their own control group. As a result, there were minimal differences in baseline characteristics and other confounding factors. Since the subjects were very similar, even the smallest effects of the intervention were clearly demonstrated, making the impact of the drug on the outcome easily measurable. Reviewing the literature revealed no studies have investigated the relationship between the change of ferritin level and inflammatory markers by using empagliflozin simultaneously. It is recommended to conduct larger studies with a greater sample size in patients with T2D, CKD, and cardiovascular complications to investigate the effect of SGLT2 inhibitor drugs on ferritin and inflammation.

In conclusion, this study revealed that levels of ferritin and inflammatory markers like interleukin-6, CRP, and uric acid decreased after empagliflozin treatment. The data from this study highlight a significant link between SGLT2 inhibition, inflammation, and iron metabolism. To investigate the effect of reducing ferritin in prediabetes and diabetic patients, it is suggested to screen for iron reservation in patients taking empagliflozin, especially diabetic patients with CKD due to reduced kidney function and erythropoiesis. We can also mention the beneficial effects of this drug in reducing inflammation, which is a major underlying cause of many complications in diabetic patients. Starting this medication in prediabetic patients at risk can help prevent the early onset of diabetes and the associated complications. This approach can also help avoid the high financial, physical, and mental costs imposed on both the patient and the healthcare system as a result of diabetes-related complications.

## Figures and Tables

**Table 1:** Baseline characteristics of patients before and after taking empagliflozin.

**Variable**	**Before treatment**	**After treatment**	**p** ** value**
Weight (kg)⁣^∗^	77.65 ± 15.97	74.5 ± 15.03	< 0.001
Systolic blood pressure (mmHg)	136.45 ± 15.37	132.68 ± 18.21	0.06
Diastolic blood pressure (mmHg)	82.31 ± 10.29	83.70 ± 9.61	0.13
BMI (kg/m^2^)	29.66 ± 4.19	28.47 ± 2.94	0.65
Waist circumstances (cm)	101.79 ± 9.11	99.35 ± 7.95	0.42

*Note:* Data are expressed as mean ± SD.

Abbreviation: BMI, body mass index.

⁣^∗^*p* < 0.05.

**Table 2:** Comparison of glycemic indices and renal function before and after taking empagliflozin.

**Variable**	**Before treatment**	**After treatment**	**p** ** value**
FPG (mg/dL)⁣^∗^	115.11 ± 28.7	106.68 ± 26.58	0.04
Hb A1c (%)⁣^∗^	6.53 ± 0.68	6.38 ± 0.75	0.03
BUN (mg/dL)	25.43 ± 6.124	26.15 ± 5.85	0.35
Cr (mg/dL)⁣^∗^	0.80 ± 0.15	0.88 ± 0.14	<0.001
GFR (mL/min)⁣^∗^	108.21 ± 31.03	94.36 ± 3.86	<0.001

*Note:* Data are expressed as mean ± SD.

Abbreviations: BUN, blood urea nitrogen; Cr, creatinine; FPG, fasting plasma glucose; GFR, glomerular filtration rate; HbA1c, glycated hemoglobin.

⁣^∗^*p* < 0.05.

**Table 3:** Comparison of changes in inflammatory markers and hematopoiesis before and after taking empagliflozin.

**Variable**	**Before treatment**	**After treatment**	**p** ** value**
Iron (*μ*mol/L)	116.27 ± 48.01	107.63 ± 38.86	0.27
TIBC⁣^∗^	343.79 ± 35.28	355.09 ± 40.56	0.01
Transferrin saturation (%)	34.51 ± 15.44	30.94 ± 12.31	0.12
Ferritin (*μ*g/L)⁣^∗^	142.38 ± 115.47	117.68 ± 94.53	0.02
CRP⁣^∗^	2.81 ± 3.82	2 ± 1.53	0.02
IL-6⁣^∗^	17.57 ± 6.63	6.52 ± 2.21	< 0.001
Uric acid (mg/dL)⁣^∗^	7.1 ± 2.32	5.66 ± 2.22	< 0.001

*Note:* Data are expressed as mean ± SD.

Abbreviations: CRP, C-reactive protein; TIBC, total iron binding capacity.

⁣^∗^p < 0.05.

**Table 4:** Correlation between serum ferritin level and serum iron level, TIBC, and transferrin saturation before and after taking empagliflozin.

**Variable**	**β**	**p** ** value**
Iron (*μ*mol/L)⁣^∗^	0.44	0.02
TIBC⁣^∗^	−0.82	< 0.001
Transferrin saturation⁣^∗^	1.86	0.002

⁣^∗^*p* < 0.05.

**Table 5:** Correlation between serum ferritin level and inflammatory factors before and after consumption of empagliflozin.

**Variable**	**β**	**p** ** value**
CRP (mg/L)	1.36	0.22
IL-6 (pg/mL)⁣^∗^	0.663	0.04
Uric acid (mg/dL)⁣^∗^	6.32	0.03

⁣^∗^*p* < 0.05.

## Data Availability

Data is available on request from the authors.
